# Portosystemic Hepatic Encephalopathy Scores (PHES) differ between Danish and German healthy populations despite their geographical and cultural similarities

**DOI:** 10.1007/s11011-024-01380-1

**Published:** 2024-07-17

**Authors:** Mette Munk Lauridsen, Lea Ladegaard Grønkjær, Jeppe Holm Atkins, Stine Ulrik Mikkelsen, Tintin Svensson, Nina Kimer, Hartmut Hecker, Gabriele Berg-Beckhoff, Karin Weissenborn, Hendrik Vilstrup

**Affiliations:** 1 Department of Gastroenterology, University Hospital of South Denmark, Finsensgade 35, 6700 Esbjerg, Denmark; 2 Gastrounit, Medical Division University Hospital Hvidovre, Kettegaards Allé 30, 2650 Hvidovre, Denmark; 3 Department of Biostatistics, Hannover Medical School, Carl-Neuberg-Straße 1, 30625 Hannover, Germany; 4 Department of Neurology, Hannover Medical School, Carl-Neuberg-Straße 1, 30625 Hannover, Germany; 5 Department of Hepatology and Gastroenterology, Aarhus University Hospital, Palle Juul-Jensens Blvd. 99, 8200 Aarhus, Denmark; 6 OPEN Open Patient Data Explorative Network, Region of Southern Denmark, J B Winsløs Vej 21, 5000 Odense, Denmark

**Keywords:** Normality tables, Liver cirrhosis, Cognition, Denmark, Hepatic encephalopathy

## Abstract

Minimal hepatic encephalopathy (MHE) is common in liver cirrhosis and is identified by psychometric tests. The portosystemic hepatic encephalopathy score (PHES) is the most widely used and serves as an inter-study comparator. PHES has not been standardised for use in the Danish population, where German normal values have been applied until now based on the notion that the populations are comparable. This study aimed to evaluate if German PHES normal values can be applied in the Danish population and establish Danish normal values if needed. 200 Danish and 217 German healthy persons underwent Number Connection Test A and B (NCT), Line Tracing Test (LTT), Digit Symbol Test (DST), and Serial Dotting Test (SDT), and based on performance, PHES was calculated. German and Danish PHES performance declined with age in all subtests but more rapidly in Danes. Both German and Danish norms were impacted by gender and education, but to a different extent in the single tests of the test battery. Accordingly, there was a need for specific Danish normal values, which are presented here. Applying the new Danish normal values instead of the German in patients with cirrhosis yielded a lower percentage of out-of-norm performances (58% vs. 66%) and, hence, a lower prevalence of MHE. Danes and Germans perform differently on PHES, and therefore, normal German values cannot be used in Danish patients. Danish normal values are presented here and yield a lower number of ‘out of norm’ performances.

**Supplementary Information:**

The online version contains supplementary material available at 10.1007/s11011-024-01380-1.

## Introduction

Finding and quantifying cognitive deficits is necessary for diagnosing minimal hepatic encephalopathy (MHE) in patients with liver cirrhosis, and psychometric tests must be used (European Association for the Study of the Liver (EASL), 2022). A few well-validated, conceptually different tests exist, but no gold standard is established (Goldbecker et al. 2013; Hansen et al. 2022; Rinčić et al. 2022). The Portosystemic Hepatic Encephalopathy Score (PHES) is endorsed as an inter-study comparator in international hepatic encephalopathy (HE) guidelines because it is widely used in many regions (Hansen et al. 2022; Vilstrup et al. 2014; Weissenborn 2001). Interpretation of the PHES requires comparison to normative data established in a healthy cohort that is representative of the socio-demographic characteristics of the target population. This includes, as a minimum, adjustment for age and, where applicable, also for gender and education. The original 1999 age-corrected German normative data have, thus far, been used in Denmark based on the notion that the test performances are comparable in such generally similar societies. German normal values were updated in 2019. This study evaluates if the 2019 German PHES normal values are applicable to Danish cohorts by comparing PHES norms in a Danish and a German healthy person cohort and assessment of the PHES of a Danish patient group with liver cirrhosis using both, the Danish and the German norms.

## Patients and methods

### Healthy Danish persons

PHES was calculated in 200 socio-demographically well-characterized, healthy adult volunteers from different age groups, educational backgrounds, and geographical areas (rural or urban) (Table 1). One hundred seventy-five were recruited from the Region of South Denmark, and 25 from Zealand. The first 100 persons were recruited between 2014 and 2016. Another 100 persons were included in 2020–2022. The sample size was based on experiences from other similar studies aiming to validate the PHES (Amodio et al. 2008; Badea et al. 2016, 2015; Coskun et al. 2017; Duarte-Rojo et al. 2011; Li et al. 2013; Seo et al. 2012; Thanapirom et al. 2023; Wunsch et al. 2013). The exclusion criteria which none fulfilled were age under 18 years, chronic liver disease or Charlson comorbidity score above 3 (Charlson et al. 1987), use of psychoactive medication, organic brain disease (e.g., prior cerebral stroke or dementia), alcohol use above 7 (female) or 14 (male) units per week, and chronic sleep disorder. We aimed to create a normal population cohort representative of the target patient group with cirrhosis and, therefore, included most middle-aged males with relatively little education.

**Table 1:** Characteristics of 200 healthy Danish adults and 217 healthy Germans who defined the German 2019 PHES normal values

Demographics	Danish *N* = 200	German *n* = 217
Gender, n (%)		
* Male*	129 (64.5)	97 (44.7)
* Female*	71 (35.5)	120 (55.3)
Age at inclusion (years), median (range)	56.0 (20–82)	47.0 (20–81)
Formal education (years), median (range)	12.5 (7–19)	10.0 (8–13)
Education group, n (%)		
≤ *9 years*	10 (5.0)	36 (16.6)
* 10–12 years*	90 (45.0)	114 (52.5)
≥ *12 years*	100 (50.0)	67 (30.9)
< *13 years*	100 (50.0)	150 (69.1)
* 13–19 years*	100 (50.0)	67 (30.9)
Profession, n (%)		
* Blue collar*	126 (63)	-
* White collar*	74 (37)	-
PSE subtest - crude results		
NCT-A (sec), mean (sd)	31.0 (14.7)	25.6 (10.1)
NCT-B (sec), mean (sd)	72.2 (27.9)	65.7 (27.8)
LTT-Time (sec), mean (sd)	68.9 (25.7)	76.2 (23.5)
LTT-error, mean (sd)	34.7 (25.7)	30.2 (25.9)
DST (boxed filled), mean (sd)	46.2 (10.6)	51.8 (11.1)
SDT (sec), mean (sd)	42.3 (11.5)	40.2 (9.7)

Participants underwent psychometric testing between 8.00 and 14.00 in an undisturbed location by one of three trained operators. All participants gave informed written consent, and The Regional Scientific Ethical Committee for Southern Denmark approved the study protocol (Protocol number S-20120196 and S-20180127).

The Danish norm data were compared to the German norm population established in Hannover in 2019, including 217 socio-demographically well-characterized, healthy adults (Table 1).

### Portosystemic hepatic encephalopathy score (PHES, Fig. 1)

The PHES is calculated based on performance in a paper-pencil test battery of 5 subtests: the number connection test A (NCT-A), number connection test B (NCT-B), line tracing test (LTT), digit symbol test (DST) and the serial dotting test (SDT). The test measures the patient’s attention, psychomotor speed and accuracy, and visuospatial perception (Weissenborn 2008). Completing the test takes approx. ten minutes, and scoring of the five subtests takes another 5 min. A short video introduction can be found here https://youtu.be/FOFmxcIYAO4. The test scoring is done by converting the time spent (in seconds) on NCT-A and B, LTT and SDT, and the number of boxes correctly filled in the DST into a number between -3 and 1, e.g., if the time spent is between -1 SD and 1 SD compared to the respective norms then a score of zero is assigned, if it is between -1 and -2 SD a score of -1 is assigned. Results better than the mean plus 1 SD are scored with 1. Two scores are assigned to the LTT — one for time spent and one for the number of errors made. The latter is evaluated using a transparent scaffold placed on top of the test sheet and manually assigning error points for each section where the line drawn by the patient is outside the designated path. The Psychometric Hepatic Encephalopathy Score (PHES) is the sum score and ranges from -18 to 6. According to German norms, a PHES below -4 is indicative of MHE in a patient with liver disease and no other cause for cognitive impairment. The test is widely used and well-validated. Training effects are mitigated by using four different test versions at repeated measurements. Our trained staff only used the original German PSE-Syndrome Test and strictly followed the German test procedure.

## Statistical analysis

The normal distribution was chosen to derive norm limits for the PSE subtests. However, since the distributions were all right- or left-skewed and, moreover, dependent on covariables, the original scores had to be transformed to achieve a normal distribution. Then, in the transformed scale, the dependency on covariables was adjusted for by covariance analysis with age as covariable and sex and formal education as cofactors. In order to be applicable, the residuals in the transformed scale should be normally distributed with a standard deviation independent of the covariables values (homoscedasticity). To achieve this, data were transformed into the logarithmic scale, except for the LTT errors, where normal distribution was achieved by square root transformation, and the SDT, where log-log transformation was used. The normality was checked by comparing the frequencies of the residuals within the ranges ± 1, 2, and 3 SD with the values of the standardized normal distribution (Chi-Square test). The homoscedasticity was checked by Levine’s test of variance homogeneity.

Negative subtest scoring points (-1, -2, -3) were assigned for residual values outside of 1, 2 and 3 standard deviations into the direction of worse performance, and  +1 for values outside of 1 standard deviation into better performance. Else, the scoring point was set to 0. The PSE sum score was calculated as the sum of all subtest points. After the elimination of outliers, this sum score was approximately normally distributed. The cut-off point for classification as abnormal was set to Mean – 2 * SD with the parameters of the PHES distribution. For the Danish population, the cut-off between normal and abnormal results is ≤ -4.

## Results

### Cohort compositions

Table 1 shows the characteristics of the two norm populations. As intended, the Danish population included more men, and the age distribution was heavy around 40–60 years, while the German age distribution was almost equal across ages 20–80. Further, in the Danish population, 50% had an education length of < 12 years, while this was true for almost 70% of the German population.

### Effect of age, gender, and educational level in Danes (Table 2)

**Table 2:** The Danish (*n* = 200) linear regression equations for each subtest used for PHES calculation. The data were all log-transformed to achieve normal distribution, except for the LTT errors, which were square root transformed, and the SDT, which was log-log transformed. The data demonstrates the impact of age, sex, and education on each subtest

Subtest	Constant	Age	Sex	Education	Standard deviation of residuals	Interpretation
			Female vs male	13–19 years vs others		
NCTA	2.63	0.015	-0.123	-0.214	0.373	NCT A time increases with age Females are faster than males People with longer education are faster
NCTB	3.61	0.014	-0.129	-0.224	0.266	NCT B time increases with age Females are faster than males People with longer education are faster
LTT time	3.84	0.007	0	-0.134	0.309	LTT time increases with age There is no effect of sex People with longer education are faster
LTT error	3.25	0.039	0	0	1.963	LT errors increase with age There is no effect sex and education
DST^a^	4.13	-0.007	0.123	0.136	0.167	Fewer boxes are filled with increasing age Females and people with longer education fill more boxes
SDT	1.25	0.002	0	- 0.041^b^ - 0.054	0.053	SDT time increases with age There is no effect of sex People with longer education are faster

Abbreviations: Portosystemic Hepatic Encephalopathy Score (PHES), Number Connection Test A (NCT-A), Number Connection Test B (NCT-B), Line Tracing Test time (LTT time), Line Tracing Test errors (LTT errors), Digit Symbol Test (DST), Serial Dotting Test (SDT)

^a^Operational sign is opposite the other subtests because DST is better the higher it gets (boxes filled)

^b^10–12 years versus others

In the Danish cohort, age had an effect on all subtests, education had an effect on NCTA, NCT B, LTT time, DST, and SDT, while sex had an effect only on NCT A, NCT B, and DST. The effect of age was most pronounced in LTT errors, NTC A, and NCT B. Danish females were approximately 12% faster than Danish males on NCTA and NCTB, and they also performed better in DST. Danes with long formal education performed better in all subtests except the LTT errors, where there was no educational effect.

Age also affected all subtests in the German cohort. Education affected NCT B, SDT, and DST, and gender affected DST (data not shown).

### Comparison between German and Danish age-dependent PHES (Table 3, Supplementary Fig. 1 & 2)

**Table 3:** Comparison between German and Danish age-dependent PHES performance. The data were all log-transformed to achieve normal distribution, except for the LTT errors, which were square root transformed, and the SDT, which was log-log transformed

PSE subtest	Regression parameter	German	Danish	(*P*-Value)	Interpretation
		Parameter values			
NCT A	a (constant):	2.641	2.369	0.040	Danes are slower at older age and show a higher standard deviation than Germans
	b (regression slope):	0.011	0.017	0.009	
	s (standard deviation):	0.282	0.374	< 0.001	
NCT B	a (constant):	3.530	3.351	n.s	No difference in age-dependent performance between the two cohorts
	b (regression slope):	0.012	0.016	n.s	
	s (standard deviation):	0.296	0.289	n.s	
LTT time	a (constant):	4.132	3.697	< 0.001	Faster completion times for Danes, especially for low and medium age
	b (regression slope):	0.003	0.008	0.024	
	s (standard deviation):	0.278	0.310	n.s	
LTT errors	a (constant):	2.559	3.257	n.s	No difference in age-dependent performance between the two cohorts
	b (regression slope):	0.051	0.039	n.s	
	s (standard deviation):	1.965	1.963	n.s	
DST	a (constant):	4.284	4.321	n.s	Slight tendency of faster age dependent decline of the number of boxes in Danes
	b (regression slope):	-0.007	- 0.009	0.022	
	s (standard deviation):	0.170	0.176	n.s	
SDT	a (constant)	1.232	1.190	0.035	Faster completion time in the lower age groups, and longer completion times in the higher age groups of Danes vs Germans. Higher standard deviation for Danes
	b (regression slope):	0.001	0.002	0.035	
	s (standard deviation):	0.042	0.055	0.004	

Abbreviations: Portosystemic Hepatic Encephalopathy Score (PHES), n.s. non-significant, Number Connection Test A (NCT-A), Number Connection Test B (NCT-B), Line Tracing Test time (LTT time), Line Tracing Test errors (LTT errors), Digit Symbol Test (DST), Serial Dotting Test (SDT)

In the NCT-A, Danes were slower than the Germans with ages above 40 (constant *p* < 0.04, slope *p* = 0.009), and their performance variation was higher (*p* < 0.001). There was no difference in the age-dependent NCT-B performance. Danes were faster in the LTT time score, especially in the lower ages (constant *p* < 0.001, slope *p* = 0.024). There was no difference in LTT errors. In the DST, there seemed to be a steeper age-dependent decline in the number of boxes filled (slope *p* = 0.022). Lastly, in the SDT, faster completion time was observed in the lower age groups, while there were longer completion times in the higher age groups of Danes than Germans (constant *p* = 0.035, slope *p* = 0.035). Further, the variation was larger in the Danish population (*p* = 0.004).

### Danish PHES normal values (Supplemental Table 1)

The Danish cohort yields PHESs that are different from and, in some subtests, more variable than the German measurements. Therefore, we calculated the Danish norm values for the five subtests used to calculate PHES (Supplementary Material). All five sub-tests are corrected for age year by year, and NCT A, B, and DST are further stratified according to gender, while results are stratified by education for the NCT A, B, LLT-time, DST, and SDT. The education strata include 13–19 years of education compared to < 12 years, except in the SDT, where three strata are necessary: 7–9 years, 10–12 years, and ≥ 13 years.

### Application of Danish and German norms to PSE-Syndrome-Test results of 122 patients with liver cirrhosis

While there was a good correlation between the patients’ PHES according to Danish and German norms, on principle, they were inconsistent for a significant number of patients (*n* = 11) (Table 4). Ten patients achieved a score < -4 applying the German norms, while their PHES was in the normal range according to Danish norms, and 1 patient would have been scored abnormal using Danish norms while his result was within the normal range according to German norms (*p* = 0.012).

**Table 4:** Cross-tabulation of PHES in a cohort of 122 Danes with liver cirrhosis using Danish normal data or German normal data. The difference is statistically significant by the McNemar test, *p* = 0.012

	n	Danish normal values		
		Normal	Abnormal	Overall (%)
German normal values	Normal	41	1	42 (34)
	Abnormal	10	70	80 (66)
	Overall (%)	51 (42)	71(58)	122 (100)

## Discussion

We found that although Germany and Denmark are neighbouring countries with similar cultural and demographic characteristics, the Danish normal PSE test performances differed from that of the German cohort. As a consequence of these observations, we present Danish norm values stratified for age, gender, and educational level, except for the LTT errors (Supplementary Table 1). These normal values are recommended for Danish cohorts, and the need for them illustrates that even culturally and geographically similar regions cannot assume the same normal values.

It is important to have accurate norms for any psychometric test, especially when the test deviation is used to diagnose conditions with treatable compromised cognition, such as MHE. We evaluated how using the new Danish PHES norms would impact MHE diagnosis in 122 patients with liver cirrhosis who had previously undergone testing and PHES calculation using the German norms (Supplementary Table 2, Table 4). Applying the Danish norms rather than the German ones resulted in a diagnostic change in 11 patients (9%). One patient went from having a normal PHES (according to German norms) to an abnormal PHES (according to Danish norms). Ten patients would have been scored abnormal according to German norms, but unimpaired according to Danish norms. As such, the Danish normal values caused the below-norm performances to decrease from 66% (80/122) to 58% (71/122). The use of German PHES norms in Danish patients has previously resulted in low to moderate prediction sensitivity of 40% for HE episodes requiring hospital admission (Wernberg et al. 2019). Using the norms presented here will perhaps result in an improved PHES test statistic.

The limitations of our study are that data were collected during an 8-year time period, and this may introduce a Flynn effect, i.e., a rise in cognitive abilities from one generation to the next (Dickinson & Hiscock 2011). However, the discrepancies in PHES test performance we observed are not likely explained by a Flynn effect because the German and Danish cohorts were established within the same decade, and we observed no difference in performances among patients recruited in 2014 versus 2022. Another limitation is that the vast majority of healthy Danish participants were recruited from a single average-sized city in Denmark but may not be representative of the Danish population as a whole. Thus, we cannot conclude on the degree of granularity appropriate for the PHES norms. Likewise, although our sample size is similar to that of other PHES validation studies, a bigger sample size would have yielded more accurate estimates. Therefore, as we continue to expand and renew the healthy cohort, a focus will be on adding people from other Danish regions.

## Conclusion

Danish and German PHES norms differ significantly regarding important population-specific characteristics. Danish normal values adequately adjusted for age, sex, and education are presented here. In a cirrhosis population, the new Danish norms reduced the out-of-norm performance percentage from 66 to 58%. Our findings illustrate the dependence of PHES subtests on subtle differences in socio-demographic factors and the need to establish regional normal values.

**Fig. 1:**
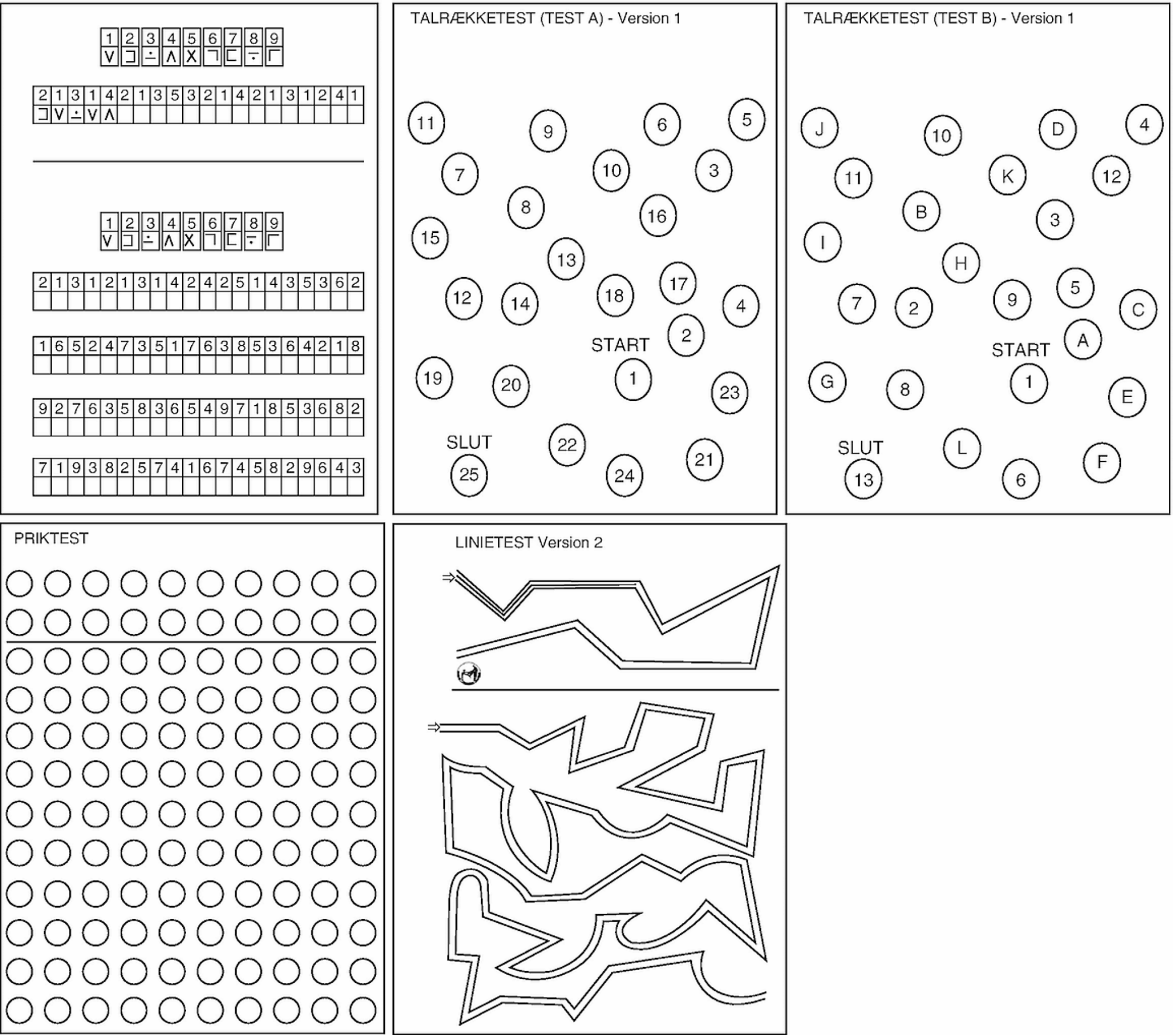
The five subtests used to calculate the Portosystemic Hepatic Encephalopathy Score (PHES). The test material (here shown in Danish) can be obtained from Hannover Medical School via the Neurometabolic study group (ag-weissenborn@mh-hannover.de). On the top left is the Digit Symbol Test (DST), the top middle is the Number Connection Test A (NCT-A), the top right is the Number Connection Test B, the bottom left is the Serial Dotting Test (SDT), and bottom right is the Line Tracing Test

### Electronic supplementary material

Below is the link to the electronic supplementary material.

Supplementary Material 1

Supplementary Material 2

Supplementary Material 3

Supplementary Material 4

## Data Availability

Source data is available upon request to first author.
