# Supplementary figures and images for: Portosystemic Hepatic Encephalopathy Scores (PHES) differ between Danish and German healthy populations despite their geographical and cultural similarities
Source: Metab Brain Dis. 2024 Jul 17;39(6):1149–55. doi: 10.1007/s11011-024-01380-1 (PMC11349773; doi:10.1007/s11011-024-01380-1)

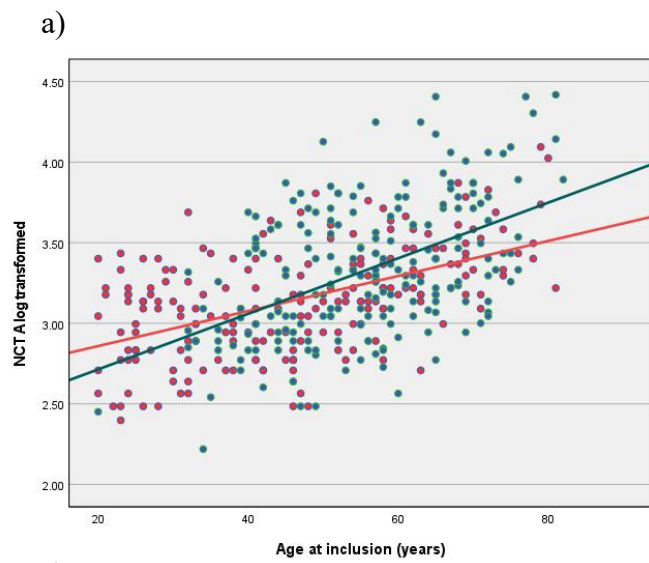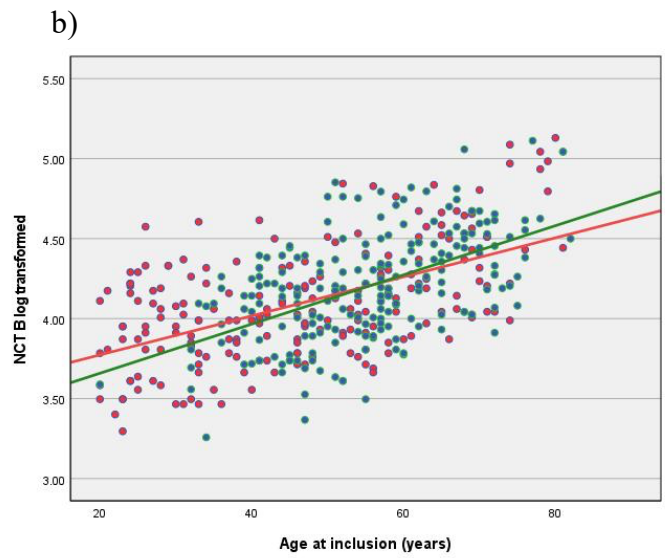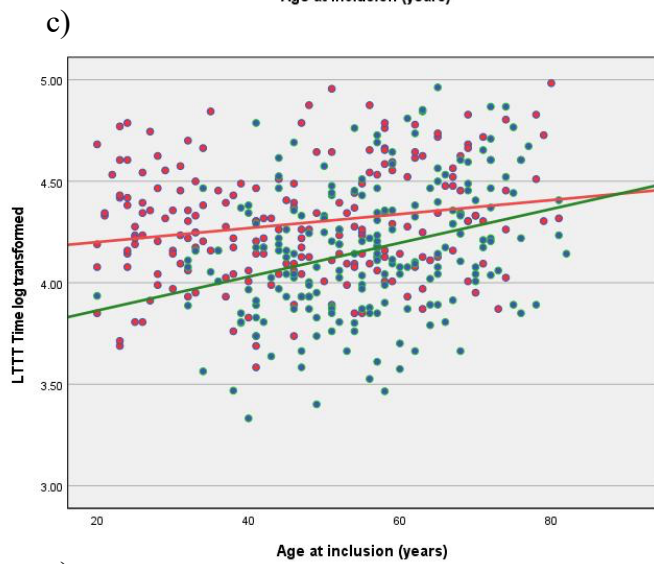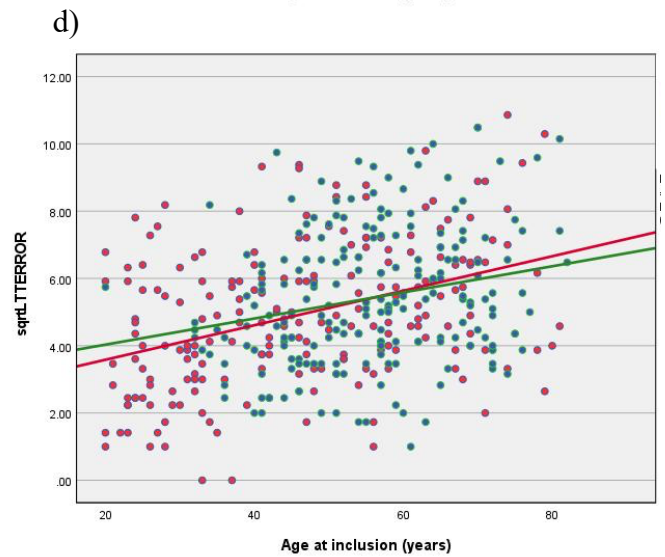

e)

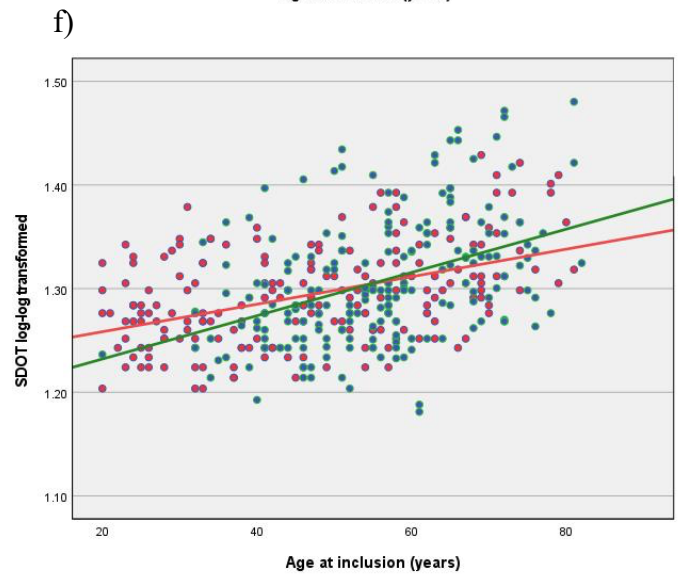

Supplement: Supplementary file 1 — Supplementary Material 1 [file 11011_2024_1380_MOESM1_ESM.pdf]
