# Supplementary material for: Portosystemic Hepatic Encephalopathy Scores (PHES) differ between Danish and German healthy populations despite their geographical and cultural similarities
Source: Metab Brain Dis. 2024 Jul 17;39(6):1149–55. doi: 10.1007/s11011-024-01380-1 (PMC11349773; doi:10.1007/s11011-024-01380-1)

Supplementary Figure 2:

Distributional plots for a) NCTA, b) NCTB, c) LTT time, d) LTT errors, e) DST, and f) SDT in German (top) and Danish (bottom) patients.

a)

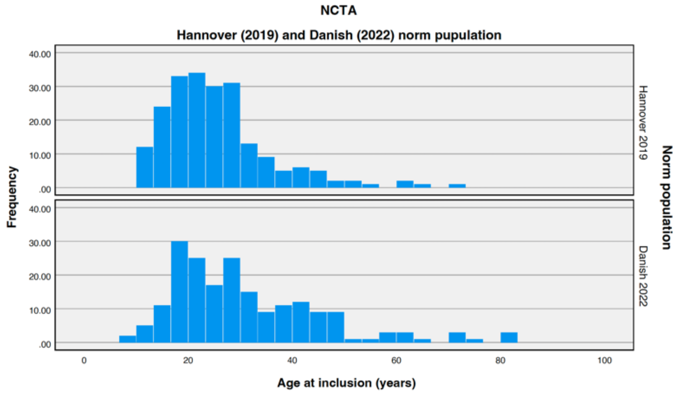

b)

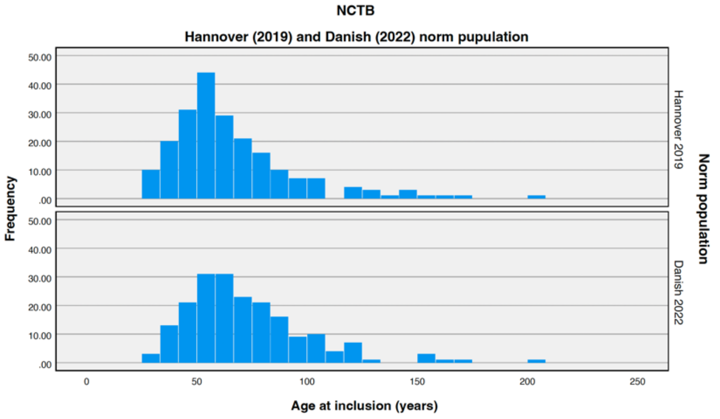

c)

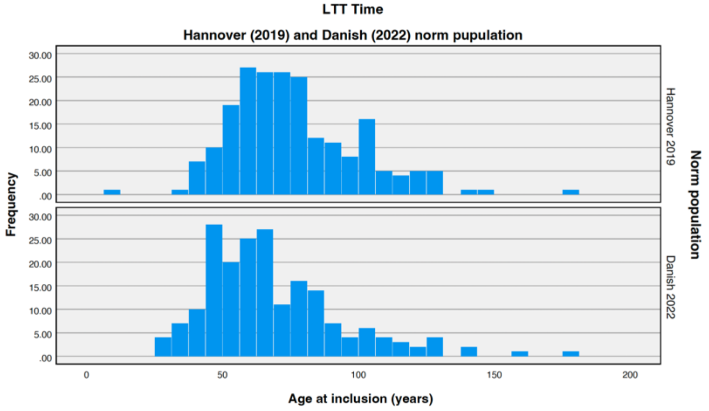

d)

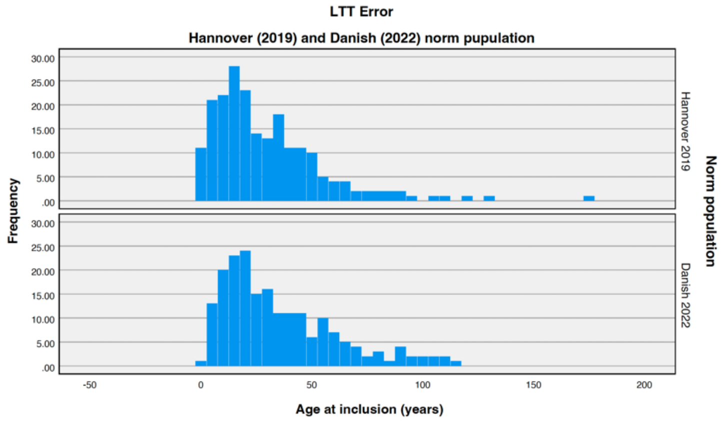

e)

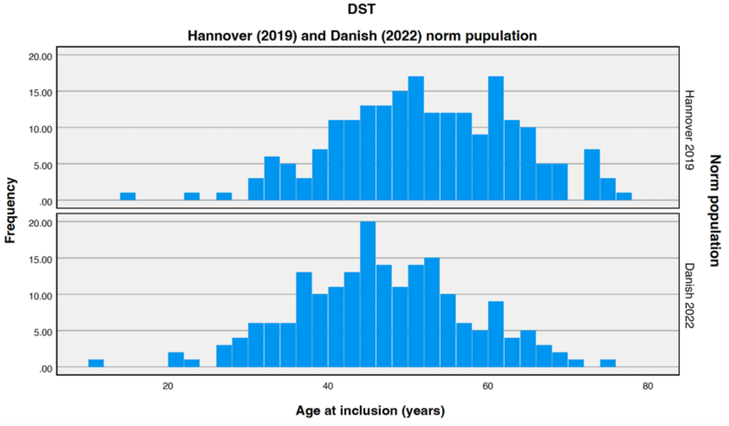

f)

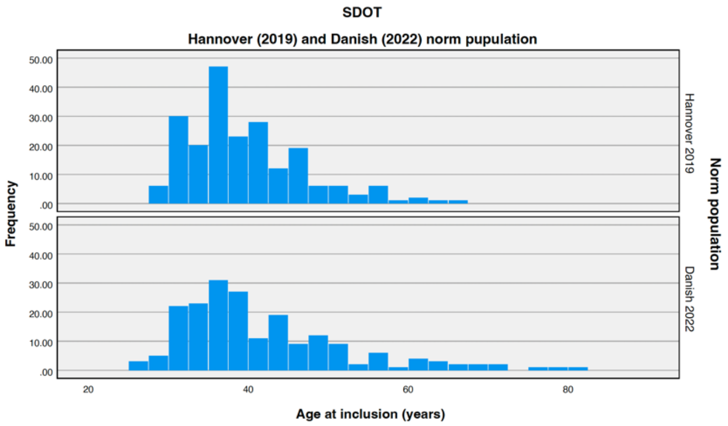

Supplement: Supplementary file 2 — Supplementary Material 2 [file 11011_2024_1380_MOESM2_ESM.pdf]
