# Supplementary material for: Portosystemic Hepatic Encephalopathy Scores (PHES) differ between Danish and German healthy populations despite their geographical and cultural similarities
Source: Metab Brain Dis. 2024 Jul 17;39(6):1149–55. doi: 10.1007/s11011-024-01380-1 (PMC11349773; doi:10.1007/s11011-024-01380-1)
Supplement: Supplementary file 3 — Supplementary Material 3 [file 11011_2024_1380_MOESM3_ESM.pdf]

Supplementary Table 1

NCTA Norm PSE Danish Data 2021, by Age and Covariables

Gender	age	NCTA: mean -1 sd, by age	NCTA: mean by age	NCTA: mean + 1 sd, by age	NCTA: mean + 2 sd, by age	NCTA: mean + 3 sd, by age
Male Formal Education: 7-12 years	30	15	22	32	47	68
	31	15	22	33	48	69
	32	15	23	33	48	70
	33	15	23	34	49	71
	34	16	23	34	50	72
	35	16	24	35	51	73
	36	16	24	35	51	74
	37	16	24	36	52	75
	38	17	25	37	53	77
	39	17	25	37	54	78
	40	17	26	38	55	79
	41	17	26	38	55	80
	42	18	26	39	56	81
	43	18	27	39	57	83
	44	18	27	40	58	84
	45	19	28	41	59	85
	46	19	28	41	60	87
	47	19	29	42	61	88
	48	19	29	43	62	89
	49	20	29	43	63	91
	50	20	30	44	64	92
	51	20	30	45	65	93
	52	21	31	45	66	95
	53	21	31	46	67	96
	54	21	32	47	68	98
	55	22	32	47	69	99
	56	22	33	48	70	101
	57	22	33	49	71	102
	58	23	34	50	72	104
	59	23	34	50	73	106
	60	24	35	51	74	107
	61	24	35	52	75	109
	62	24	36	53	76	111
	63	25	37	54	78	112
	64	25	37	54	79	114
	65	25	38	55	80	116
	66	26	38	56	81	118
	67	26	39	57	82	119
	68	27	39	58	84	121
	69	27	40	59	85	123
	70	28	41	60	86	125
	71	28	41	61	88	127
	72	28	42	61	89	129
	73	29	43	62	90	131
	74	29	43	63	92	133
	75	30	44	64	93	135
	76	30	45	65	95	137
	77	31	45	66	96	139
	78	31	46	67	98	141
	79	32	47	68	99	144
	80	32	47	69	101	146
	81	33	48	71	102	148
	82	33	49	72	104	150
Male						

Formal Education: 13 - 19 years	30	12	18	26	38	55
	31	12	18	27	38	56
	32	12	18	27	39	56
	33	12	19	27	40	57
	34	12	19	28	40	58
	35	13	19	28	41	59
	36	13	19	29	41	60
	37	13	20	29	42	61
	38	13	20	30	43	62
	39	14	20	30	43	63
	40	14	21	31	44	64
	41	14	21	31	45	65
	42	14	21	31	45	66
	43	14	22	32	46	67
	44	15	22	32	47	68
	45	15	22	33	48	69
	46	15	23	33	48	70
	47	15	23	34	49	71
	48	16	23	34	50	72
	49	16	24	35	51	73
	50	16	24	36	51	74
	51	16	24	36	52	76
	52	17	25	37	53	77
	53	17	25	37	54	78
	54	17	26	38	55	79
	55	17	26	38	55	80
	56	18	26	39	56	82
	57	18	27	40	57	83
	58	18	27	40	58	84
	59	19	28	41	59	85
	60	19	28	41	60	87
	61	19	29	42	61	88
	62	19	29	43	62	89
	63	20	29	43	63	91
	64	20	30	44	64	92
	65	20	30	45	65	94
	66	21	31	45	66	95
	67	21	31	46	67	97
	68	21	32	47	68	98
	69	22	32	47	69	100
	70	22	33	48	70	101
	71	22	33	49	71	103
	72	23	34	50	72	104
	73	23	34	50	73	106
	74	24	35	51	74	107
	75	24	35	52	75	109
	76	24	36	53	76	111
	77	25	37	54	78	113
	78	25	37	54	79	114
	79	25	38	55	80	116
	80	26	38	56	81	118
	81	26	39	57	83	120
	82	27	40	58	84	122
Female Formal Education: 7-12 years	30	13	19	29	41	60
	31	13	20	29	42	61
	32	13	20	30	43	62
	33	14	20	30	43	63
	34	14	21	31	44	64
	35	14	21	31	45	65
	36	14	21	31	45	66
	37	14	22	32	46	67

	38	15	22	32	47	68
	39	15	22	33	48	69
	40	15	23	33	48	70
	41	15	23	34	49	71
	42	16	23	34	50	72
	43	16	24	35	51	73
	44	16	24	36	51	74
	45	16	24	36	52	75
	46	17	25	37	53	77
	47	17	25	37	54	78
	48	17	26	38	55	79
	49	17	26	38	55	80
	50	18	26	39	56	81
	51	18	27	40	57	83
	52	18	27	40	58	84
	53	19	28	41	59	85
	54	19	28	41	60	87
	55	19	29	42	61	88
	56	19	29	43	62	89
	57	20	29	43	63	91
	58	20	30	44	64	92
	59	20	30	45	65	94
	60	21	31	45	66	95
	61	21	31	46	67	96
	62	21	32	47	68	98
	63	22	32	47	69	99
	64	22	33	48	70	101
	65	22	33	49	71	103
	66	23	34	50	72	104
	67	23	34	50	73	106
	68	24	35	51	74	107
	69	24	35	52	75	109
	70	24	36	53	76	111
	71	25	37	54	78	112
	72	25	37	54	79	114
	73	25	38	55	80	116
	74	26	38	56	81	118
	75	26	39	57	83	120
	76	27	39	58	84	121
	77	27	40	59	85	123
	78	28	41	60	86	125
	79	28	41	61	88	127
	80	28	42	61	89	129
	81	29	43	62	90	131
	82	29	43	63	92	133
Female Formal Education: 13 - 19 years	30	10	16	23	34	49
	31	10	16	24	34	49
	32	11	16	24	35	50
	33	11	16	24	35	51
	34	11	17	25	36	52
	35	11	17	25	36	52
	36	11	17	25	37	53
	37	12	17	26	37	54
	38	12	18	26	38	55
	39	12	18	27	38	56
	40	12	18	27	39	57
	41	12	19	27	40	57
	42	12	19	28	40	58
	43	13	19	28	41	59
	44	13	19	29	42	60
	45	13	20	29	42	61
	46	13	20	30	43	62
	47	14	20	30	43	63

48	14	21	31	44	64
49	14	21	31	45	65
50	14	21	31	46	66
51	14	22	32	46	67
52	15	22	32	47	68
53	15	22	33	48	69
54	15	23	33	48	70
55	15	23	34	49	71
56	16	23	34	50	72
57	16	24	35	51	73
58	16	24	36	51	74
59	16	25	36	52	76
60	17	25	37	53	77
61	17	25	37	54	78
62	17	26	38	55	79
63	17	26	38	56	80
64	18	26	39	56	82
65	18	27	40	57	83
66	18	27	40	58	84
67	19	28	41	59	85
68	19	28	41	60	87
69	19	29	42	61	88
70	20	29	43	62	89
71	20	30	43	63	91
72	20	30	44	64	92
73	20	30	45	65	94
74	21	31	45	66	95
75	21	31	46	67	97
76	21	32	47	68	98
77	22	32	47	69	100
78	22	33	48	70	101
79	22	33	49	71	103
80	23	34	50	72	104
81	23	34	50	73	106
82	24	35	51	74	108

NCT B Norm PSE Danish Data 2021, by Age and Covariables

Gender	age	NCTB: mean -1 sd, by age	NCTB: mean by age	NCTB: mean + 1 sd, by age	NCTB: mean + 2 sd, by age	NCTB: mean + 3 sd, by age
Male Formal Education: 7-12 years	30	42	56	74	97	126
	31	43	56	75	98	127
	32	43	57	76	99	129
	33	44	58	77	100	131
	34	45	59	78	102	132
	35	45	60	79	103	134
	36	46	60	80	105	136
	37	46	61	81	106	138
	38	47	62	83	107	140
	39	48	63	84	109	142
	40	48	64	85	110	144
	41	49	65	86	112	145
	42	50	66	87	113	147
	43	50	66	88	115	149
	44	51	67	89	116	151

	45	52	68	91	118	153
	46	53	69	92	119	156
	47	53	70	93	121	158
	48	54	71	94	123	160
	49	55	72	96	124	162
	50	55	73	97	126	164
	51	56	74	98	128	166
	52	57	75	99	129	169
	53	58	76	101	131	171
	54	59	77	102	133	173
	55	59	78	104	135	175
	56	60	79	105	137	178
	57	61	80	106	138	180
	58	62	81	108	140	183
	59	63	83	109	142	185
	60	64	84	111	144	188
	61	64	85	112	146	190
	62	65	86	114	148	193
	63	66	87	115	150	195
	64	67	88	117	152	198
	65	68	89	118	154	201
	66	69	91	120	156	203
	67	70	92	122	158	206
	68	71	93	123	160	209
	69	72	94	125	163	212
	70	73	96	127	165	215
	71	74	97	128	167	218
	72	75	98	130	169	220
	73	76	100	132	171	223
	74	77	101	133	174	226
	75	78	102	135	176	230
	76	79	104	137	179	233
	77	80	105	139	181	236
	78	81	107	141	183	239
	79	82	108	143	186	242
	80	83	110	145	188	245
	81	85	111	147	191	249
	82	86	113	149	193	252
Male Formal Education: 13 - 19 years	30	34	45	60	77	101
	31	34	45	60	78	102
	32	35	46	61	79	103
	33	35	46	62	81	105
	34	35	47	63	82	106
	35	36	48	64	83	107
	36	36	48	65	84	109
	37	37	49	65	85	110
	38	37	50	66	86	112
	39	38	50	67	87	113
	40	39	51	68	88	115
	41	39	52	69	90	116
	42	40	52	70	91	118
	43	40	53	71	92	120
	44	41	54	72	93	121
	45	41	55	73	94	123
	46	42	55	74	96	125
	47	42	56	75	97	126
	48	43	57	76	98	128
	49	44	58	77	100	130
	50	44	58	78	101	131
	51	45	59	79	102	133
	52	45	60	80	104	135
	53	46	61	81	105	137
	54	47	62	82	106	139

	55	47	62	83	108	140
	56	48	63	84	109	142
	57	49	64	85	111	144
	58	49	65	86	112	146
	59	50	66	88	114	148
	60	51	67	89	115	150
	61	51	68	90	117	152
	62	52	69	91	118	154
	63	53	70	92	120	156
	64	54	71	94	122	158
	65	54	71	95	123	161
	66	55	72	96	125	163
	67	56	73	97	127	165
	68	57	74	99	128	167
	69	57	75	100	130	169
	70	58	76	101	132	172
	71	59	78	103	134	174
	72	60	79	104	135	176
	73	61	80	105	137	179
	74	61	81	107	139	181
	75	62	82	108	141	184
	76	63	83	110	143	186
	77	64	84	111	145	189
	78	65	85	113	147	191
	79	66	86	114	149	194
	80	67	88	116	151	196
	81	67	89	117	153	199
	82	68	90	119	155	202
Female Formal Education: 7-12 years	30	37	49	65	85	110
	31	38	50	66	86	112
	32	38	50	67	87	113
	33	39	51	68	88	115
	34	39	52	69	90	117
	35	40	52	70	91	118
	36	40	53	71	92	120
	37	41	54	72	93	121
	38	41	55	73	95	123
	39	42	55	74	96	125
	40	42	56	75	97	126
	41	43	57	76	98	128
	42	44	58	77	100	130
	43	44	58	78	101	131
	44	45	59	79	102	133
	45	45	60	80	104	135
	46	46	61	81	105	137
	47	47	62	82	107	139
	48	47	62	83	108	141
	49	48	63	84	109	142
	50	49	64	85	111	144
	51	49	65	86	112	146
	52	50	66	88	114	148
	53	51	67	89	115	150
	54	51	68	90	117	152
	55	52	69	91	119	154
	56	53	70	92	120	156
	57	54	71	94	122	159
	58	54	72	95	123	161
	59	55	73	96	125	163
	60	56	73	97	127	165
	61	57	74	99	128	167
	62	57	76	100	130	170
	63	58	77	101	132	172
	64	59	78	103	134	174
	65	60	79	104	136	177

	66	61	80	106	137	179
	67	61	81	107	139	181
	68	62	82	108	141	184
	69	63	83	110	143	186
	70	64	84	111	145	189
	71	65	85	113	147	191
	72	66	86	114	149	194
	73	67	88	116	151	197
	74	68	89	117	153	199
	75	68	90	119	155	202
	76	69	91	121	157	205
	77	70	92	122	159	207
	78	71	94	124	161	210
	79	72	95	126	163	213
	80	73	96	127	166	216
	81	74	98	129	168	219
	82	75	99	131	170	222
Female Formal Education: 13 - 19 years	30	29	39	53	68	89
	31	30	40	53	69	90
	32	30	40	54	70	91
	33	31	41	55	71	92
	34	31	41	55	72	93
	35	32	42	56	73	95
	36	32	42	57	74	96
	37	32	43	58	75	97
	38	33	44	58	76	98
	39	33	44	59	77	100
	40	34	45	60	78	101
	41	34	45	61	79	103
	42	35	46	62	80	104
	43	35	47	62	81	105
	44	36	47	63	82	107
	45	36	48	64	83	108
	46	37	49	65	84	110
	47	37	49	66	85	111
	48	38	50	67	87	113
	49	38	51	68	88	114
	50	39	51	68	89	116
	51	39	52	69	90	117
	52	40	53	70	91	119
	53	40	53	71	93	120
	54	41	54	72	94	122
	55	42	55	73	95	124
	56	42	56	74	96	125
	57	43	56	75	98	127
	58	43	57	76	99	129
	59	44	58	77	100	130
	60	44	59	78	102	132
	61	45	59	79	103	134
	62	46	60	80	104	136
	63	46	61	81	106	138
	64	47	62	82	107	139
	65	48	63	83	109	141
	66	48	64	85	110	143
	67	49	65	86	111	145
	68	50	65	87	113	147
	69	50	66	88	115	149
	70	51	67	89	116	151
	71	52	68	90	118	153
	72	52	69	92	119	155
	73	53	70	93	121	157
	74	54	71	94	122	159
	75	55	72	95	124	162

76	55	73	97	126	164
77	56	74	98	127	166
78	57	75	99	129	168
79	58	76	101	131	170
80	58	77	102	133	173
81	59	78	103	134	175
82	60	79	105	136	177

LTT Time Norm PSE Danish Data 2021, by Age and Covariables

Formal Education	age	LTTTime: mean -1 sd, by age	LTTTime: mean by age	LTTTime: mean + 1 sd, by age	LTTTime: mean + 2 sd, by age	LTTTime: mean + 3 sd, by age
Formal Education: 7-12 years	30	41	57	78	107	145
	31	42	58	79	107	146
	32	42	58	79	108	147
	33	42	58	80	109	148
	34	43	59	81	110	149
	35	43	59	81	110	150
	36	43	60	82	111	151
	37	44	60	82	112	152
	38	44	60	83	113	154
	39	44	61	83	114	155
	40	44	61	84	114	156
	41	45	62	85	115	157
	42	45	62	85	116	158
	43	45	63	86	117	159
	44	46	63	86	118	160
	45	46	63	87	118	161
	46	46	64	88	119	162
	47	47	64	88	120	163
	48	47	65	89	121	165
	49	47	65	89	122	166
	50	48	66	90	123	167
	51	48	66	91	123	168
	52	48	67	91	124	169
	53	49	67	92	125	170
	54	49	68	93	126	172
	55	49	68	93	127	173
	56	50	68	94	128	174
	57	50	69	95	129	175
	58	50	69	95	130	176
	59	51	70	96	130	178
	60	51	70	97	131	179
	61	52	71	97	132	180
	62	52	71	98	133	181
	63	52	72	99	134	183
	64	53	72	99	135	184
	65	53	73	100	136	185
	66	53	73	101	137	187
	67	54	74	101	138	188
	68	54	74	102	139	189
	69	55	75	103	140	190
	70	55	76	103	141	192
	71	55	76	104	142	193
	72	56	77	105	143	194
	73	56	77	106	144	196

	74	56	78	106	145	197
	75	57	78	107	146	199
	76	57	79	108	147	200
	77	58	79	109	148	201
	78	58	80	109	149	203
	79	58	80	110	150	204
	80	59	81	111	151	206
	81	59	82	112	152	207
	82	60	82	112	153	208
Formal Education: 13 - 19 years	30	36	50	69	93	127
	31	36	50	69	94	128
	32	37	51	70	95	129
	33	37	51	70	95	130
	34	37	51	71	96	131
	35	37	52	71	97	131
	36	38	52	71	97	132
	37	38	52	72	98	133
	38	38	53	72	99	134
	39	39	53	73	99	135
	40	39	54	73	100	136
	41	39	54	74	101	137
	42	39	54	75	101	138
	43	40	55	75	102	139
	44	40	55	76	103	140
	45	40	55	76	104	141
	46	40	56	77	104	142
	47	41	56	77	105	143
	48	41	57	78	106	144
	49	41	57	78	106	145
	50	42	57	79	107	146
	51	42	58	79	108	147
	52	42	58	80	109	148
	53	43	59	80	109	149
	54	43	59	81	110	150
	55	43	59	82	111	151
	56	43	60	82	112	152
	57	44	60	83	113	153
	58	44	61	83	113	154
	59	44	61	84	114	155
	60	45	62	84	115	156
	61	45	62	85	116	158
	62	45	62	86	116	159
	63	46	63	86	117	160
	64	46	63	87	118	161
	65	46	64	87	119	162
	66	47	64	88	120	163
	67	47	65	89	121	164
	68	47	65	89	121	165
	69	48	66	90	122	167
	70	48	66	90	123	168
	71	48	66	91	124	169
	72	49	67	92	125	170
	73	49	67	92	126	171
	74	49	68	93	127	172
	75	50	68	94	127	174
	76	50	69	94	128	175
	77	50	69	95	129	176
	78	51	70	96	130	177
	79	51	70	96	131	179
	80	51	71	97	132	180
	81	52	71	98	133	181
	82	52	72	98	134	182

LTT Error Norm PSE Danish Data 2021, by Age

age	LTTError: mean -1 sd, by age	LTTError: mean by age	LTTError: mean + 1 sd, by age	LTTError: mean + 2 sd, by age	LTTError: mean + 3 sd, by age
30	6	20	41	70	107
31	6	20	42	71	108
32	6	20	42	71	108
33	6	21	43	72	109
34	6	21	43	73	110
35	7	21	44	73	111
36	7	22	44	74	112
37	7	22	45	75	113
38	7	22	45	75	113
39	7	23	46	76	114
40	8	23	46	77	115
41	8	24	47	78	116
42	8	24	47	78	117
43	8	24	48	79	117
44	9	25	49	80	118
45	9	25	49	80	119
46	9	25	50	81	120
47	9	26	50	82	121
48	9	26	51	82	122
49	10	27	51	83	123
50	10	27	52	84	123
51	10	27	52	84	124
52	10	28	53	85	125
53	11	28	53	86	126
54	11	29	54	87	127
55	11	29	55	87	128
56	12	30	55	88	129
57	12	30	56	89	130
58	12	30	56	90	130
59	12	31	57	90	131
60	13	31	58	91	132
61	13	32	58	92	133
62	13	32	59	92	134
63	13	33	59	93	135
64	14	33	60	94	136
65	14	33	60	95	137
66	14	34	61	95	138
67	15	34	62	96	139
68	15	35	62	97	139
69	15	35	63	98	140
70	16	36	64	99	141
71	16	36	64	99	142
72	16	37	65	100	143
73	17	37	65	101	144
74	17	38	66	102	145
75	17	38	67	102	146
76	18	39	67	103	147
77	18	39	68	104	148
78	18	40	69	105	149
79	19	40	69	106	150
80	19	40	70	106	151
81	19	41	70	107	152
82	20	41	71	108	153

DST Norm PSE Danish Data 2021, by Age and Covariables

Gender	age	DST: mean + 1 sd, by age	DST: mean by age	DST: mean - 1 sd, by age	DST: mean - 2 sd, by age	DST: mean -3 sd, by age
Male Formal Education: 7-12 years	30	59	50	42	35	30
	31	59	49	41	35	29
	32	58	49	41	35	29
	33	58	49	41	34	29
	34	57	48	40	34	29
	35	57	48	40	34	28
	36	57	47	40	33	28
	37	56	47	39	33	28
	38	56	47	39	33	28
	39	55	46	39	33	28
	40	55	46	38	32	27
	41	54	46	38	32	27
	42	54	45	38	32	27
	43	54	45	38	32	27
	44	53	45	37	31	27
	45	53	44	37	31	26
	46	52	44	37	31	26
	47	52	44	36	31	26
	48	52	43	36	30	26
	49	51	43	36	30	25
	50	51	43	36	30	25
	51	50	42	35	30	25
	52	50	42	35	30	25
	53	50	42	35	29	25
	54	49	41	34	29	25
	55	49	41	34	29	24
	56	49	41	34	29	24
	57	48	40	34	28	24
	58	48	40	33	28	24
	59	47	40	33	28	24
	60	47	39	33	28	23
	61	47	39	33	27	23
	62	46	39	32	27	23
	63	46	39	32	27	23
	64	46	38	32	27	23
	65	45	38	32	27	22
	66	45	38	31	26	22
	67	45	37	31	26	22
	68	44	37	31	26	22
	69	44	37	31	26	22
	70	44	36	30	26	22
	71	43	36	30	25	21
	72	43	36	30	25	21
	73	43	36	30	25	21
	74	42	35	29	25	21
	75	42	35	29	25	21
	76	42	35	29	24	21
	77	41	35	29	24	20
	78	41	34	29	24	20
	79	41	34	28	24	20
	80	40	34	28	24	20
	81	40	34	28	23	20

	82	40	33	28	23	20
Male Formal Education: 13 - 19 years	30	68	57	48	40	34
	31	67	57	47	40	34
	32	67	56	47	40	33
	33	66	56	47	39	33
	34	66	55	46	39	33
	35	65	55	46	39	33
	36	65	54	46	38	32
	37	64	54	45	38	32
	38	64	54	45	38	32
	39	63	53	44	38	32
	40	63	53	44	37	31
	41	62	52	44	37	31
	42	62	52	43	37	31
	43	61	52	43	36	31
	44	61	51	43	36	30
	45	60	51	42	36	30
	46	60	50	42	36	30
	47	60	50	42	35	30
	48	59	50	41	35	30
	49	59	49	41	35	29
	50	58	49	41	34	29
	51	58	48	40	34	29
	52	57	48	40	34	29
	53	57	48	40	34	28
	54	56	47	40	33	28
	55	56	47	39	33	28
	56	56	47	39	33	28
	57	55	46	39	33	27
	58	55	46	38	32	27
	59	54	46	38	32	27
	60	54	45	38	32	27
	61	54	45	37	32	27
	62	53	45	37	31	26
	63	53	44	37	31	26
	64	52	44	37	31	26
	65	52	43	36	31	26
	66	52	43	36	30	26
	67	51	43	36	30	25
	68	51	42	35	30	25
	69	50	42	35	30	25
	70	50	42	35	29	25
	71	50	42	35	29	25
	72	49	41	34	29	24
	73	49	41	34	29	24
	74	48	41	34	29	24
	75	48	40	34	28	24
	76	48	40	33	28	24
	77	47	40	33	28	23
	78	47	39	33	28	23
	79	47	39	33	27	23
	80	46	39	32	27	23
	81	46	38	32	27	23
	82	46	38	32	27	23
Female Formal Education: 7-12 years	30	67	56	47	40	34
	31	66	56	47	39	33
	32	66	55	46	39	33
	33	65	55	46	39	33
	34	65	55	46	39	33

	35	64	54	45	38	32
	36	64	54	45	38	32
	37	63	53	45	38	32
	38	63	53	44	37	32
	39	63	52	44	37	31
	40	62	52	44	37	31
	41	62	52	43	36	31
	42	61	51	43	36	31
	43	61	51	43	36	30
	44	60	50	42	36	30
	45	60	50	42	35	30
	46	59	50	42	35	30
	47	59	49	41	35	29
	48	58	49	41	35	29
	49	58	49	41	34	29
	50	57	48	40	34	29
	51	57	48	40	34	28
	52	57	47	40	33	28
	53	56	47	39	33	28
	54	56	47	39	33	28
	55	55	46	39	33	28
	56	55	46	38	32	27
	57	54	46	38	32	27
	58	54	45	38	32	27
	59	54	45	38	32	27
	60	53	45	37	31	27
	61	53	44	37	31	26
	62	52	44	37	31	26
	63	52	44	36	31	26
	64	52	43	36	30	26
	65	51	43	36	30	25
	66	51	43	36	30	25
	67	50	42	35	30	25
	68	50	42	35	30	25
	69	50	42	35	29	25
	70	49	41	34	29	24
	71	49	41	34	29	24
	72	49	41	34	29	24
	73	48	40	34	28	24
	74	48	40	33	28	24
	75	47	40	33	28	24
	76	47	39	33	28	23
	77	47	39	33	27	23
	78	46	39	32	27	23
	79	46	39	32	27	23
	80	46	38	32	27	23
	81	45	38	32	27	22
	82	45	38	31	26	22
Female Formal Education: 13 - 19 years	30	77	64	54	46	39
	31	76	64	54	45	38
	32	76	63	53	45	38
	33	75	63	53	45	38
	34	74	63	52	44	37
	35	74	62	52	44	37
	36	73	62	52	44	37
	37	73	61	51	43	36
	38	72	61	51	43	36
	39	72	60	50	43	36
	40	71	60	50	42	36
	41	71	59	50	42	35
	42	70	59	49	42	35
	43	69	58	49	41	35
	44	69	58	48	41	35

	45	68	57	48	41	34
	46	68	57	48	40	34
	47	67	57	47	40	34
	48	67	56	47	40	33
	49	66	56	47	39	33
	50	66	55	46	39	33
	51	65	55	46	39	33
	52	65	54	46	38	32
	53	64	54	45	38	32
	54	64	54	45	38	32
	55	63	53	44	38	32
	56	63	53	44	37	31
	57	62	52	44	37	31
	58	62	52	43	37	31
	59	61	52	43	36	31
	60	61	51	43	36	30
	61	60	51	42	36	30
	62	60	50	42	36	30
	63	60	50	42	35	30
	64	59	50	41	35	30
	65	59	49	41	35	29
	66	58	49	41	34	29
	67	58	48	40	34	29
	68	57	48	40	34	29
	69	57	48	40	34	28
	70	56	47	40	33	28
	71	56	47	39	33	28
	72	56	47	39	33	28
	73	55	46	39	33	27
	74	55	46	38	32	27
	75	54	46	38	32	27
	76	54	45	38	32	27
	77	53	45	37	32	27
	78	53	44	37	31	26
	79	53	44	37	31	26
	80	52	44	37	31	26
	81	52	43	36	31	26
	82	51	43	36	30	26

SDOT Norm PSE Danish Data 2021, by Age and Covariables

Formal Education	age	SDOT: mean -1 sd, by age	SDOT: mean by age	SDOT: mean + 1 sd, by age	SDOT: mean + 2 sd, by age	SDOT: mean + 3 sd, by age
7 - 9 years	30	32	40	49	61	76
	31	33	40	50	61	77
	32	33	40	50	62	77
	33	33	41	50	62	78
	34	33	41	51	63	78
	35	33	41	51	63	79
	36	34	42	51	64	80
	37	34	42	52	64	80
	38	34	42	52	65	81
	39	34	42	52	65	82
	40	35	43	53	66	82
	41	35	43	53	66	83
	42	35	43	54	67	84
	43	35	44	54	67	84

	44	36	44	54	68	85
	45	36	44	55	68	86
	46	36	44	55	69	86
	47	36	45	56	69	87
	48	36	45	56	70	88
	49	37	45	56	70	88
	50	37	46	57	71	89
	51	37	46	57	71	90
	52	37	46	58	72	91
	53	38	47	58	72	91
	54	38	47	59	73	92
	55	38	47	59	74	93
	56	39	48	59	74	94
	57	39	48	60	75	94
	58	39	48	60	75	95
	59	39	49	61	76	96
	60	40	49	61	76	97
	61	40	49	62	77	98
	62	40	50	62	78	98
	63	40	50	63	78	99
	64	41	50	63	79	100
	65	41	51	63	80	101
	66	41	51	64	80	102
	67	42	52	64	81	103
	68	42	52	65	81	103
	69	42	52	65	82	104
	70	42	53	66	83	105
	71	43	53	66	83	106
	72	43	53	67	84	107
	73	43	54	67	85	108
	74	44	54	68	85	109
	75	44	55	68	86	110
	76	44	55	69	87	111
	77	44	55	70	88	112
	78	45	56	70	88	113
	79	45	56	71	89	114
	80	45	57	71	90	115
	81	46	57	72	90	115
	82	46	57	72	91	116
10 - 12 years	30	28	34	42	52	64
	31	28	35	43	52	64
	32	28	35	43	52	65
	33	29	35	43	53	65
	34	29	35	43	53	66
	35	29	36	44	54	66
	36	29	36	44	54	67
	37	29	36	44	54	67
	38	30	36	45	55	68
	39	30	36	45	55	68
	40	30	37	45	56	69
	41	30	37	46	56	70
	42	30	37	46	56	70
	43	31	37	46	57	71
	44	31	38	46	57	71
	45	31	38	47	58	72
	46	31	38	47	58	72
	47	31	38	47	58	73
	48	32	39	48	59	73
	49	32	39	48	59	74
	50	32	39	48	60	75
	51	32	39	49	60	75
	52	32	40	49	61	76
	53	33	40	49	61	76
	54	33	40	50	62	77
	55	33	41	50	62	78
	56	33	41	51	62	78

	57	33	41	51	63	79
	58	34	41	51	63	79
	59	34	42	52	64	80
	60	34	42	52	64	81
	61	34	42	52	65	81
	62	35	43	53	65	82
	63	35	43	53	66	83
	64	35	43	53	66	83
	65	35	43	54	67	84
	66	35	44	54	67	85
	67	36	44	55	68	85
	68	36	44	55	68	86
	69	36	45	55	69	87
	70	36	45	56	69	87
	71	37	45	56	70	88
	72	37	46	57	70	89
	73	37	46	57	71	90
	74	37	46	57	72	90
	75	38	47	58	72	91
	76	38	47	58	73	92
	77	38	47	59	73	93
	78	38	48	59	74	93
	79	39	48	60	74	94
	80	39	48	60	75	95
	81	39	49	61	76	96
	82	39	49	61	76	96
13 - 19 years	30	27	33	40	49	61
	31	27	33	41	50	61
	32	27	33	41	50	62
	33	27	33	41	50	62
	34	28	34	41	51	63
	35	28	34	42	51	63
	36	28	34	42	51	63
	37	28	34	42	52	64
	38	28	35	42	52	64
	39	28	35	43	52	65
	40	29	35	43	53	65
	41	29	35	43	53	66
	42	29	35	44	54	66
	43	29	36	44	54	67
	44	29	36	44	54	67
	45	30	36	45	55	68
	46	30	36	45	55	68
	47	30	37	45	55	69
	48	30	37	45	56	69
	49	30	37	46	56	70
	50	31	37	46	57	71
	51	31	38	46	57	71
	52	31	38	47	58	72
	53	31	38	47	58	72
	54	31	38	47	58	73
	55	31	39	48	59	73
	56	32	39	48	59	74
	57	32	39	48	60	74
	58	32	39	49	60	75
	59	32	40	49	61	76
	60	33	40	49	61	76
	61	33	40	50	61	77
	62	33	41	50	62	77
	63	33	41	50	62	78
	64	33	41	51	63	79
	65	34	41	51	63	79
	66	34	42	52	64	80
	67	34	42	52	64	81
	68	34	42	52	65	81
	69	34	42	53	65	82

70	35	43	53	66	83
71	35	43	53	66	83
72	35	43	54	67	84
73	35	44	54	67	85
74	36	44	55	68	85
75	36	44	55	68	86
76	36	45	55	69	87
77	36	45	56	69	87
78	37	45	56	70	88
79	37	46	57	70	89
80	37	46	57	71	89
81	37	46	57	72	90
82	38	46	58	72	91
