# Supplementary material for: Portosystemic Hepatic Encephalopathy Scores (PHES) differ between Danish and German healthy populations despite their geographical and cultural similarities
Source: Metab Brain Dis. 2024 Jul 17;39(6):1149–55. doi: 10.1007/s11011-024-01380-1 (PMC11349773; doi:10.1007/s11011-024-01380-1)
Supplement: Supplementary file 4 — Supplementary Material 4 [file 11011_2024_1380_MOESM4_ESM.docx]

|  | Cirrhosis n=122 |
| --- | --- |
| Age Mean years (SD) | 60 (8.9) |
| Female/male % | 25/97 (21%/79%) |
| Education   - Under 13 years - Over 13 years | 28 (23%)  94 (77%) |
| MELD mean (SD) | 11.5 (4.9) |
| Previous overt HE | 33 (27%) |
